# Supplementary material for: PFN1 Inhibits Myogenesis of Bovine Myoblast Cells via Cdc42-PAK/JNK
Source: Cells. 2022 Oct 11;11(20):3188. doi: 10.3390/cells11203188 (PMC9600610; doi:10.3390/cells11203188)
Supplement: Supplementary file 1 [file cells-11-03188-s001.zip › cells-1926160-supplementary.pdf]

Table S1: Primers of gene cloning.

Genes	Primer Name	Primer Sequences（5’-3’）
PFN1	PFN1-F	CCGAAGCTTGCCACCATGGCCGGGTGGAACGCCTA
	PFN1-R	CCCGAATTCGTACTGGAACGCCGCAGGT
Cdc42	Cdc42-F	CCCAAGCTTGCCACCATGCAGACAATTAAGTGCGT
	Cdc42-R	CCGGAATTCTCATAGCAGCACACACCTGC

Note: the underlined part in the table is the restriction enzyme cleavage site.

Table S2: Sequences of si-bat-PFN1/Cdc42.

Fragment Name	Sequence (5’-3’)
si-bta-PFN1	GCAAAGACCGGTCAAGTTT
si-bta-Cdc42	CCTGAAGGCTGTCAAGTAT

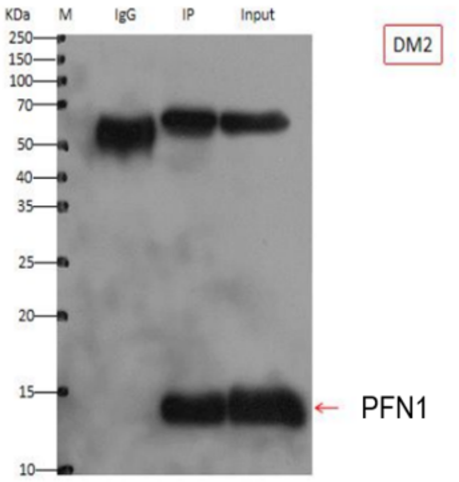

Figure S1: Detect PFN1 antibody after IP.

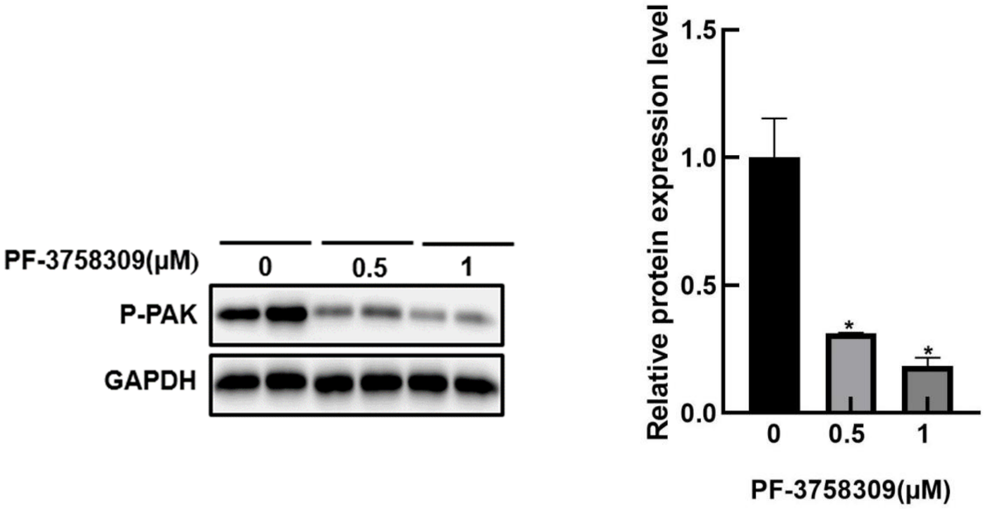

Figure S2: Screening of optimal concentrations of P-PAK inhibitors.

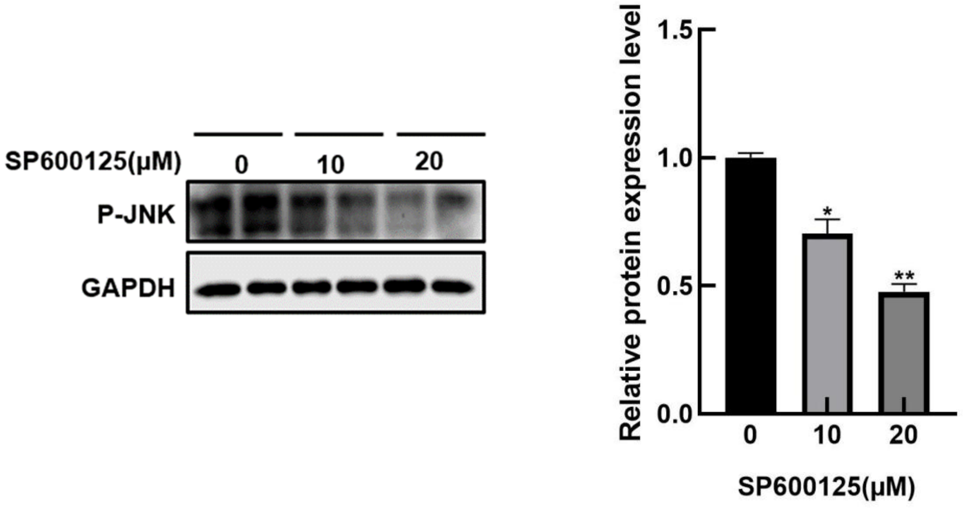

Figure S3: Screening of optimal concentrations of P-JNK inhibitors.
